# Exploiting Bulk Photovoltaic Effect in a Polar Hybrid Perovskite Towards Self‐Powered Detection of Weak Ultraviolet Polarized Light

**DOI:** 10.1002/advs.76653

**Published:** 2026-07-23

**Authors:** Xinyuan Zhang, Jianbo Wu, Wai‐Yeung Wong

**Affiliations:** ^1^ Department of Applied Biology and Chemical Technology and Research Institute For Smart Energy The Hong Kong Polytechnic University Hong Kong People's Republic of China; ^2^ The Hong Kong Polytechnic University Shenzhen Research Institute Shenzhen People's Republic of China; ^3^ Department of Materials Science and Engineering City University of Hong Kong Hong Kong People's Republic of China

**Keywords:** light intensity, perovskite, photodetector, photovoltaic effect, taxonomy, ultraviolet

## Abstract

Weak light detection is essential for achieving high‐performance photodetectors with minimal power consumption; however, it remains challenging due to the high dark current under operating voltage. Herein, a self‐powered photodetector based on a polar perovskite crystal, namely (4‐AMP)PbBr_4_ (4‐AMP = 4‐(aminomethyl)‐piperidine), is reported that exhibits exceptional sensitivity to ultraweak ultraviolet (UV) polarized light. (4‐AMP)PbBr_4_ crystallizes in a polar space group of *P*ca2_1_ and generates spontaneous polarization that gives rise to a strong bulk photovoltaic effect (BPVE), serving as a driving force for spatial separation/transport of photogenerated carriers. As a result, self‐powered photodetectors using (4‐AMP)PbBr_4_ crystals exhibit significantly suppressed current to sub‐pA level in the absence of light, and can be “activated” with ultraweak UV light radiation, showcasing superior photoresponse characteristics. More interestingly, by leveraging its inherent BPVE, the resultant device exhibits intriguing polarization‐sensitive behavior along with a large anisotropic ratio of 8 at 1 µW/cm^2^, among the highest values of the reported polarized light detectors. Even at a polarized light intensity down to 60 nW/cm^2^, the anisotropic ratio can still reach 1.8. Our findings establish a promising platform for energy‐efficient, light‐driven devices with potential applications in self‐powered polarized light detection.

## Graphical abstract

Benefiting from intrinsic polarization, DJ‐type 2D perovskite crystals deliver self‐powered polarization‐resolved photocurrent under weak polarized UV light via the bulk photovoltaic effect, showing a high anisotropic factor of 8.

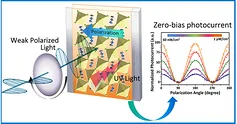

## 1 Introduction

Weak light detection is critically needed in medical and security applications, including fluorescence microscopy, radars, and quantum communication [1, 2, 3, 4]. For a high‐performance photodetector, the lowest detectable light radiation is determined by the noise, which is generally governed by dark current, although some other types of noise may be present at the same time [5, 6, 7]. Thus, it is significant to suppress the device dark current so that the signal generated at weak light radiation can be well resolved above the noise, which dictates the device sensitivity (responsivity and detectivity) [8, 9, 10]. To suppress the dark current, many of the reported photodetectors rely on using semiconducting materials with relatively high bulk resistivity [11, 12]. This is based on the Ohm's law, *J* = *U*/*ρ*, in which *J* is the dark current density, *U* stands for the electric field intensity, and *ρ* is the bulk resistivity [13]. However, the bulk resistivities of state‐of‐the‐art semiconductors such as silicon (Si), germanium (Ge), and black phosphorus (BP) are still too low to produce a low dark current, with values of about 1 µA cm^−2^ in their detectors working even at a low external electric field [14, 15, 16]. Therefore, new strategies are urgently needed to suppress the dark current for the development of high‐performance photodetectors with low detection limits.

Fabricating self‐powered photodetectors can provide a clue to address such a challenge [17, 18, 19, 20]. A conventional photoconductive‐type detector operates under a relatively high bias to generate photoresponse, which unavoidably results in a large dark current. In contrast, a self‐powered photodetector works without an external energy supply, which can lead to a low dark current coming from the built‐in electric potential [21, 22]. Recently, self‐powered photodetectors using polar semiconductors have shown great potential in this portfolio [23, 24, 25]. Polar semiconductors present a bulk photovoltaic effect (BPVE) induced by the spontaneous polarization along their polar axis [26, 27, 28, 29, 30]. The spontaneous polarization can generate a built‐in electric field within the materials and serve to separate/transport the photogenerated electron‐hole pairs without external energy supply [31, 32]. Therefore, at zero bias, short‐circuited current with low dark current can be observed in the orientation of spontaneous polarization [33]. More notably, it has been revealed that the BPVE photocurrent is highly polarization sensitive [34, 35, 36, 37], which can be explained by the tensor model established by Fridkin et al. and has been well demonstrated in some inorganic ferroelectrics (e.g. BiFeO_3_ and BaTiO_3_) [38]. Thus, constructing self‐powered photodetectors of polar semiconductors to explore their polarization sensitivity and weak light detection performance is interesting.

In the past decade, 2D organic‐inorganic hybrid perovskites (OIHPs) have delivered remarkable breakthroughs in the field of optoelectronics owing to their superior properties, including large absorption coefficient, defect tolerance, and stability [39, 40, 41]. Even more surprising is their structural flexibility [42]. The presence of large organic cations can enhance the dynamic molecular motion and enlarge the perovskite cages, making the 2D OIHP structures significantly deviate from the ideal cubic symmetry and rendering them to form polar lattices [43]. Based on the above merits, herein, we report a self‐powered photodetector using a polar 2D OIHP crystal, (4‐AMP)PbBr_4_. The resultant device shows pronounced photoresponse in the UV spectral region with an ultralow dark current of 2 × 10^−13^ A, making it possible for weak light detection. More impressively, due to the presence of BPVE, the device is highly sensitive to ultraweak polarized light, exhibiting a large anisotropy ratio from 8 to 1.8 across varied incident light intensities (from 1 µW/cm^2^ to 60 nW/cm^2^). Moreover, the devices also deliver high detectivity of 8.8 × 10^12^ Jones and a fast response time. Our work on polar OIHPs marks a step toward developing high‐performance self‐powered photodetectors with a low detection limit.

## 2 Results and Discussion

The 2D monolayered (4‐AMP)PbBr_4_ was synthesized according to previous reports, and its transparent bulk crystals with dimensions of about 2 × 2 × 1 mm^3^ were grown via a temperature‐cooling solution method (Figure S1) [44]. Figure S2a shows the X‐ray diffraction (XRD) profiles for (4‐AMP)PbBr_4_, which exhibits well‐oriented characteristic peaks with a narrow full width at half maximum (FWHM, Figure S2b) of 0.02 degrees, comparable to other high‐quality crystals (Table S1). As shown in Figure 1a, (4‐AMP)PbBr_4_ adopts a (100)‐oriented hybrid perovskite lattice with a polar space group of *P*ca2_1_. The polar structure of (4‐AMP)PbBr_4_ was confirmed by the second harmonic generation (SHG) signal (Figure S3). Structural analysis suggests that the polar axis of (4‐AMP)PbBr_4_ is along *c* axis (Figure 1b), which will generate spontaneous polarization along that direction and further satisfy the requirements of bulk photovoltaic effect (BFPV). Moreover, different from those widely studied Ruddlesden‐Popper type perovskites that rely on weak van der Waals interaction between the neighboring layers, the utilization of short diamine cation here removes the van der Waals gap and minimizes the Br···Br distance, facilitating the formation of stronger H‐bonds deep inside the inorganic framework (Figure 1c). Therefore, (4‐AMP)PbBr_4_ tends to hold a more stable structure than those Ruddlesden‐Popper type perovskites.

**FIGURE 1:**
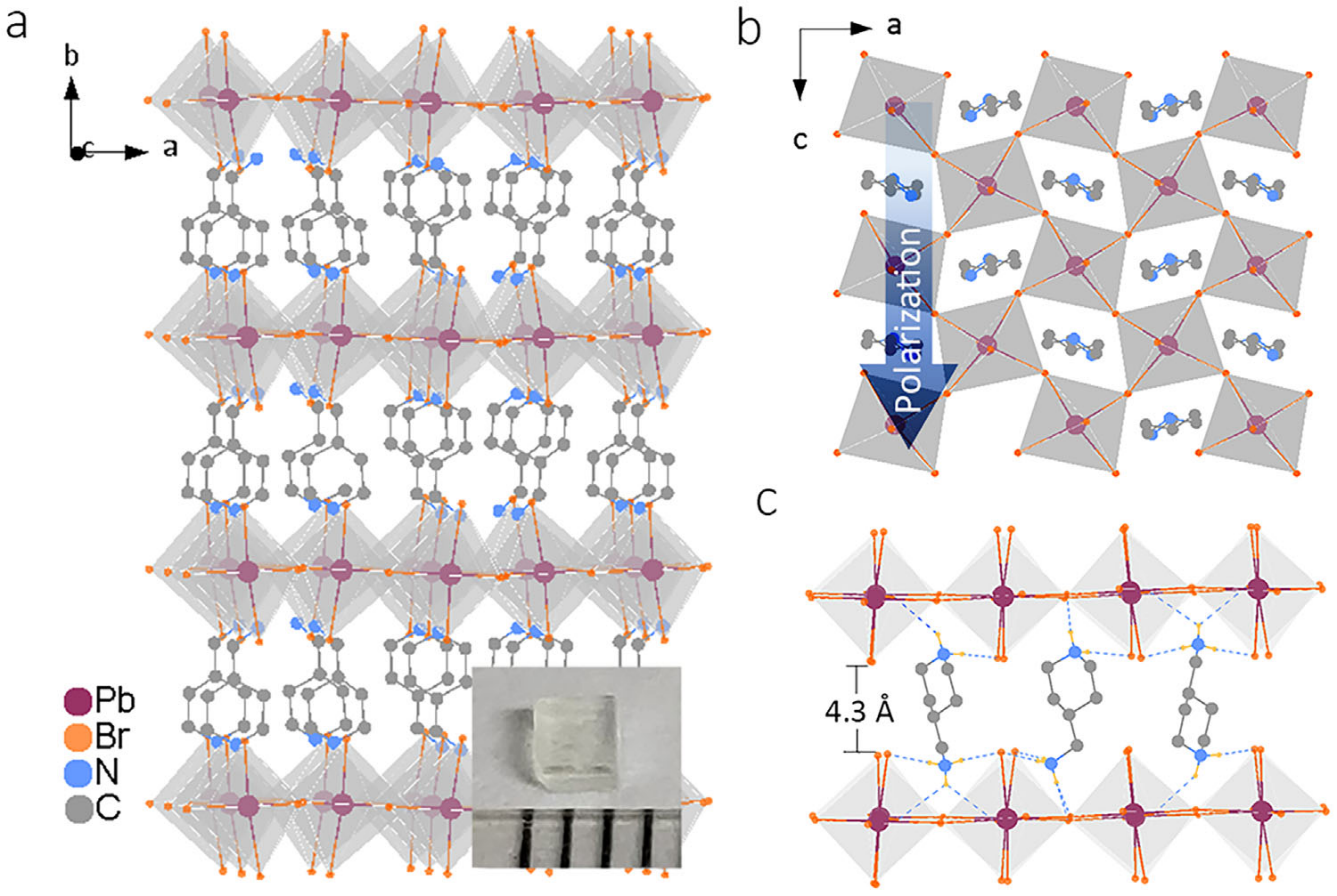
(a) Structure of the monolayered (4‐AMP)PbBr_4_. The inset is a picture of a (4‐AMP)PbBr_4_ single crystal. (b) The direction of spontaneous polarization and polar axis of (4‐AMP)PbBr_4_. (c) The H‐bonds inside the inorganic framework.

We then conducted single‐crystal X‐ray diffraction (SCXRD), atomic force microscopy (AFM), and scanning electron microscopy (SEM) measurements to study the crystalline quality of the (4‐AMP)PbBr_4_ crystals. Figure 2a exhibits the SCXRD image of the (4‐AMP)PbBr_4_ crystal along its [001] direction. Well‐aligned lattice diffraction spots are observed, indicating the high single‐crystalline quality of the grown (4‐AMP)PbBr_4_ crystals. AFM studies in Figure 2b reveal few grain boundaries, which is consistent with the SEM characterizations (Figure S4). To quantitatively estimate the trap densities (*N*_trap_) of the (4‐AMP)PbBr_4_ crystals, we performed the space‐charge‐limited current (SCLC) technique. As displayed in Figure 2c, the current‐voltage (*I*‐*V*) curve shows an ohmic response at a lower bias. At a higher bias, traps in the (4‐AMP)PbBr_4_ crystals are obviously filled with the rapid rise of current, indicating a trap‐filled limit (*TFL*) regime. The *N*_trap_ is calculated to be about 4 × 10^10^ cm^−3^, which is comparable to those of high‐quality MAPbX_3_ crystals (where X = Br, I; *N*_trap_ = 3 × 10^10^ cm^−3^) [45]. Moreover, carrier mobility and carrier diffusion length are estimated to be 0.98 cm^2^ V^−1^ s^−1^ and 0.5 µm, respectively. The above results support that the grown (4‐AMP)PbBr_4_ crystals are of favorable crystalline quality, which can potentially benefit carrier transport and thus the performance of optoelectronic devices. Furthermore, optical properties of the (4‐AMP)PbBr_4_ crystals were characterized by utilizing the ultraviolet‐visible (UV–Vis) absorption spectra and photoluminescence (PL) spectra. As shown in Figure 2d, the sharp absorption edge of (4‐AMP)PbBr_4_ is located at ∼420 nm, which endows an experimental bandgap of 2.93 eV according to the Tauc equation (Figure S5), indicating its strong optical absorption in the UV region. PL emission properties were studied in the solid state. A broad white emission is observed with the peak position at 400 nm under 375 nm laser excitation (Figure 2d), which may originate from the octahedral distortion. Besides, the PL decay lifetime is estimated to be 8.2 ns (Figure 2e). Moreover, we measured the temperature‐dependent conductivity to investigate the semiconducting properties of (4‐AMP)PbBr_4_ crystals. As shown in Figure 2f, the conductivities of the (4‐AMP)PbBr_4_ crystal along its three crystallographic axes increase rapidly as the temperature increases, indicating the typical semiconductor characteristics of (4‐AMP)PbBr_4_ crystals. Interestingly, the conductivity measured along the *c*‐axis is significantly higher than that along the *b*‐axis, which can be ascribed to the layered construction of (4‐AMP)PbBr_4_ [46].

**FIGURE 2:**
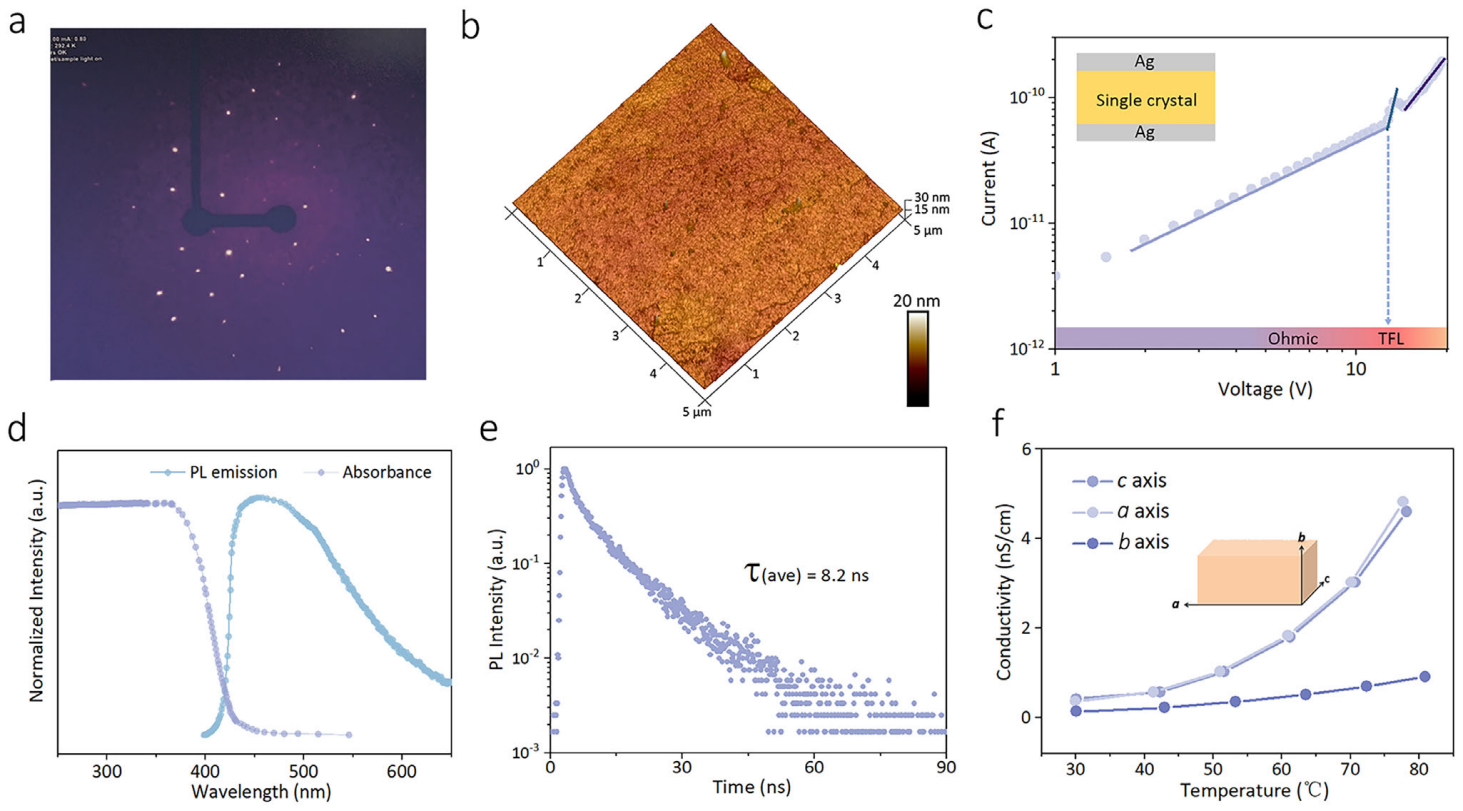
(a) SCXRD patterns of the (4‐AMP)PbBr_4_ crystal along the [001] direction. (b) AFM image of the (4‐AMP)PbBr_4_ crystals. (c) The space‐charge‐limited current of the (4‐AMP)PbBr_4_ crystal. (d) UV–vis and PL spectra of the (4‐AMP)PbBr_4_ crystals. (e) The PL lifetime of (4‐AMP)PbBr_4_ crystals. (f) Temperature‐dependent conductivity of the (4‐AMP)PbBr_4_ crystals along *a*‐, *b*‐, and *c*‐axis.

Based on the knowledge of those fundamental properties, we next turned to investigate the photodetection performance of the high‐quality (4‐AMP)PbBr_4_ crystals. Figure 3a and Figure S6 show the schematic illustration and energy band alignment diagram of the vertical‐structure devices, respectively. Considering the direction of spontaneous polarization, silver (Ag) electrodes were deposited perpendicular to the *c*‐axis of (4‐AMP)PbBr_4_ crystals, and the thicknesses of the used crystals are about 1 mm. In the present photodetectors, the (4‐AMP)PbBr_4_ crystal acts as a photoactive semiconductor to produce electron‐hole pairs under light excitation; and those photogenerated carriers are then captured by the opposite electrodes to generate photocurrent. Spectral‐dependence photocurrent of the (4‐AMP)PbBr_4_ crystal devices is measured from 377 to 785 nm, showing the peak photoresponse at 377 nm, which indicates the UV radiation detection ability of the (4‐AMP)PbBr_4_ crystal photodetectors (Figure 3b). Due to the limited bandgap of (4‐AMP)PbBr_4_ crystals, the detectors can hardly maintain efficient photocurrent at wavelengths longer than 405 nm (Figure S7). Therefore, we focused on the photodetection behaviors of the (4‐AMP)PbBr_4_ detectors in the UV range. *I–V* curves of the present devices measured under 377 nm laser illumination are shown in Figure 3c. The linear dark *I‐V* trace of (4‐AMP)PbBr_4_ indicates the Ohmic contact of the metal/perovskite interface (Figure S8). With the incident power increasing, the photocurrent of the detectors increases significantly. The dependence of the photocurrent on the light power under a bias voltage of 10 V is shown in Figure S9. The curve can be well fitted with a power law, *I_P_ ∼ P^α^*, in which α determines the device photoresponse to the light power. The fitting reveals an almost straight line with *α* = 0.81, which is smaller than an ideal device (*α* = 1), suggesting the existence of sub‐bandgap trap states in (4‐AMP)PbBr_4_ or at the electrode/(4‐AMP)PbBr_4_ interface. Notably, due to the spontaneous polarization of (4‐AMP)PbBr_4_ crystals along its *c*‐axis, the photodetectors using (4‐AMP)PbBr_4_ crystals exhibit clear BPVE with a photovoltage of ∼0.15 V (Figure S10). Such a value is comparable to many other polar 2D hybrid perovskites, such as [(*R*)‐MPA]_4_AgBiI_8_ and (BDA)(EA)_2_Pb_3_Br_10_ [34, 47]. In contrast, there is no photovoltage observed along the *a* and *b* axes (Figure 3d). This phenomenon supports the polarization direction of *c* axis, and further verifies that the photovoltage in the (4‐AMP)PbBr_4_ crystals originates from BPVE rather than metal/perovskite interfaces, as the Schottky contacts do not provide such directional dependence. The observation of BPVE implies our UV photodetectors can act as self‐powered photodetectors operating at zero bias. *I‐t* curves of the present devices under different light powers are displayed in Figure 3e. Impressively, the self‐powered photodetectors exhibit an obvious photoresponse, with obvious on/off switching ratio of ∼44 under the ultraweak UV radiation (60 nW/cm^2^). Such a high performance can be ascribed to the low dark current (about 0.29 pA) of the devices in a self‐powered mode. Typically, for perovskite photodetectors, relatively high bias is applied on the two opposite electrodes to extract the carriers to ensure charge collection efficiency, and their dark current can be high in those photoconductive‐type detectors [5]. In contrast, our device works under zero bias; the ultralow dark current in the self‐powered mode renders ultraweak light detection to be well resolved. We note that Figure 3c indicates a dark current of about 1 pA at zero bias. The discrepancy in zero‐bias dark current between *I‐t* and *I–V* measurements may originate from the different testing conditions. The *I‐V* characterization involves continuous voltage sweeping across a wide bias range; when the voltage crosses 0 V, residual trapped carriers, migrated mobile ions, together with capacitive transient current, can elevate the zero‐bias dark current captured by fast *I–V* scans. In contrast, the zero‐bias *I‐t* measurement is performed under sustained static zero bias without voltage perturbation. The sufficient relaxation time in the *I‐t* test largely alleviates the above issues, which yields a smaller dark current than that extracted from dynamic *I–V* scans. Meanwhile, as mentioned above, the carrier diffusion length of the (4‐AMP)PbBr_4_ crystals is calculated to be ∼0.5 µm, which is far shorter than the device channel length (200 µm). The distinct zero‐bias photocurrent in the present devices can be described as follows: 1) The zero‐bias photocurrent is generated by the carriers that migrate to electrodes from the light spot. Those photogenerated carriers near the electrodes are preferentially extracted. 2) The uniform built‐in electric field throughout the (4‐AMP)PbBr_4_ crystal provides a steady unidirectional driving force for the sequential separation/transport of photogenerated photocarriers. Photocarriers thus can migrate directionally to the electrodes and be thus extracted to yield the signal. Figure 3f shows that the self‐powered photodetector retains the photocurrent intensity after ∼10^2^ switching cycles, indicating its high operational stability.

**FIGURE 3:**
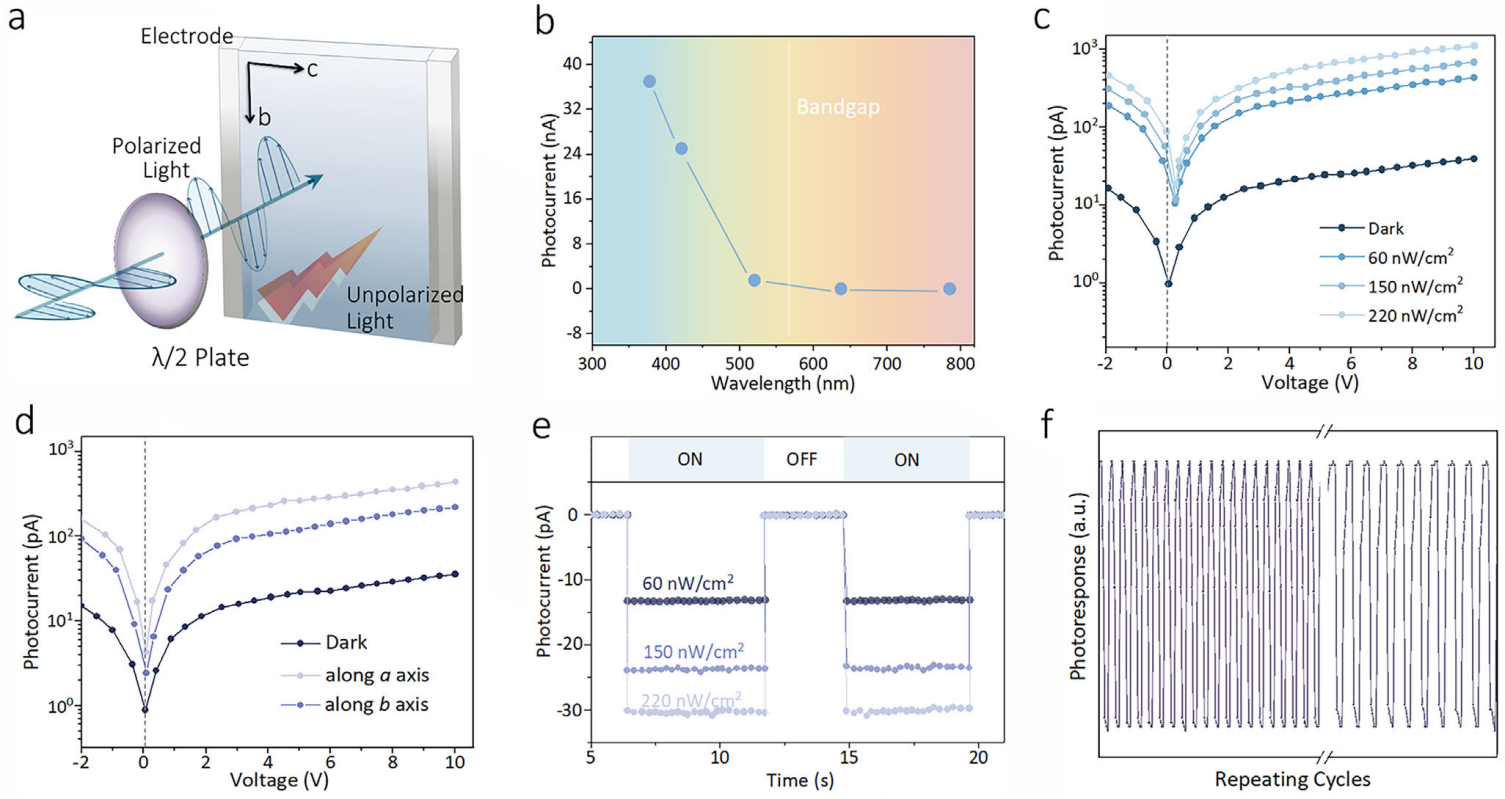
(a) Illustration of the photodetectors using the (4‐AMP)PbBr_4_ crystals. (b) Photoresponse of the (4‐AMP)PbBr_4_ crystal detectors at wavelengths ranging from 377 to 785 nm. (c) Photocurrents of the (4‐AMP)PbBr_4_ crystal devices along the *c* axis at 377 nm under different light intensities. (d) Photocurrent of the (4‐AMP)PbBr_4_ crystal devices along the *a* and *b* axes. (e) *I‐t* measurement of the on/off switching cycles of the (4‐AMP)PbBr_4_ crystal photodetectors under different light intensities. (f) On/off switching cycles of the (4‐AMP)PbBr_4_ crystal detectors at zero bias.

Another intriguing profit of BPVE is polarization sensitivity, which encourages us to further evaluate the capability of our devices for polarized light detection in the UV region at self‐powered mode. We used a half‐wave (*λ*/2) plate to modulate the light polarization by continuously rotating the angle between the fast axis of *λ*/2 plate and the incident light polarization (as mentioned in Figure 3a). The incident light radiates at the (4‐AMP)PbBr_4_ crystal, where its *c*‐axis direction (spontaneous polarization direction) is defined as 0° polarization. Notably, zero bias is applied on the electrodes during the experiments, and thus the polarization‐sensitive photoresponse is solely driven by the BPVE. Figure 4a exhibits the photovoltage response of the self‐powered (4‐AMP)PbBr_4_ crystal photodetectors at different polarization angles under the radiation of UV light. It is clear that the photovoltage value changes dramatically by modulating the polarization angles of incident light, where the maximum response is at ∼0° and ∼180°, parallel to the *c*‐axis direction; and the minimum response is at ∼90° and ∼270°, perpendicular to the *c*‐axis direction. More interestingly, such photovoltage anisotropy fully translates to a high polarization sensitivity in the photocurrent. As shown in Figure 4b, the photocurrent under parallel excitation (*I*_IIc_) is strikingly larger than that under perpendicular excitation (*I*_⊥c_), demonstrating the capability for polarized light detection. Photoresponse of the (4‐AMP)PbBr_4_ crystal detectors in the direction perpendicular to the polar axis (along the *a*‐axis) is also studied. Negligible distinction in the *a*‐axis photocurrent to the different polarization of incident light is observed (Figure S11), further supporting the zero‐bias photocurrent of (4‐AMP)PbBr_4_ originating from the BPVE [38, 48]. More notably, the (4‐AMP)PbBr_4_ crystal polarization‐sensitive photodetectors can detect weak polarized light with intensity down to 60 nW/cm^2^, with an impressive anisotropy ratio (*I*_IIc_/*I*_⊥c_) of ∼1.8 (Figure 4c). The light intensity of 60 nW/cm^2^ is significantly lower than most of the reported perovskite polarization‐sensitive photodetectors, whose detection limits are typically at the mW/cm^2^ level [49, 50, 51]. Such a high performance can be directly ascribed to the ultralow dark current of the device in self‐powered mode. The anisotropy ratio of photocurrent increases as the light intensity increases, and reaches the highest value of 8 under 1 µW/cm^2^ LPL illumination. Such a figure‐of‐merit is comparable to or even better than that of many classic UV polarized light detectors using nanowires and anisotropic 2D materials [52, 53]. Experimental anisotropy ratios of some reported polarization‐sensitive photodetectors are listed in Table S2.

**FIGURE 4:**
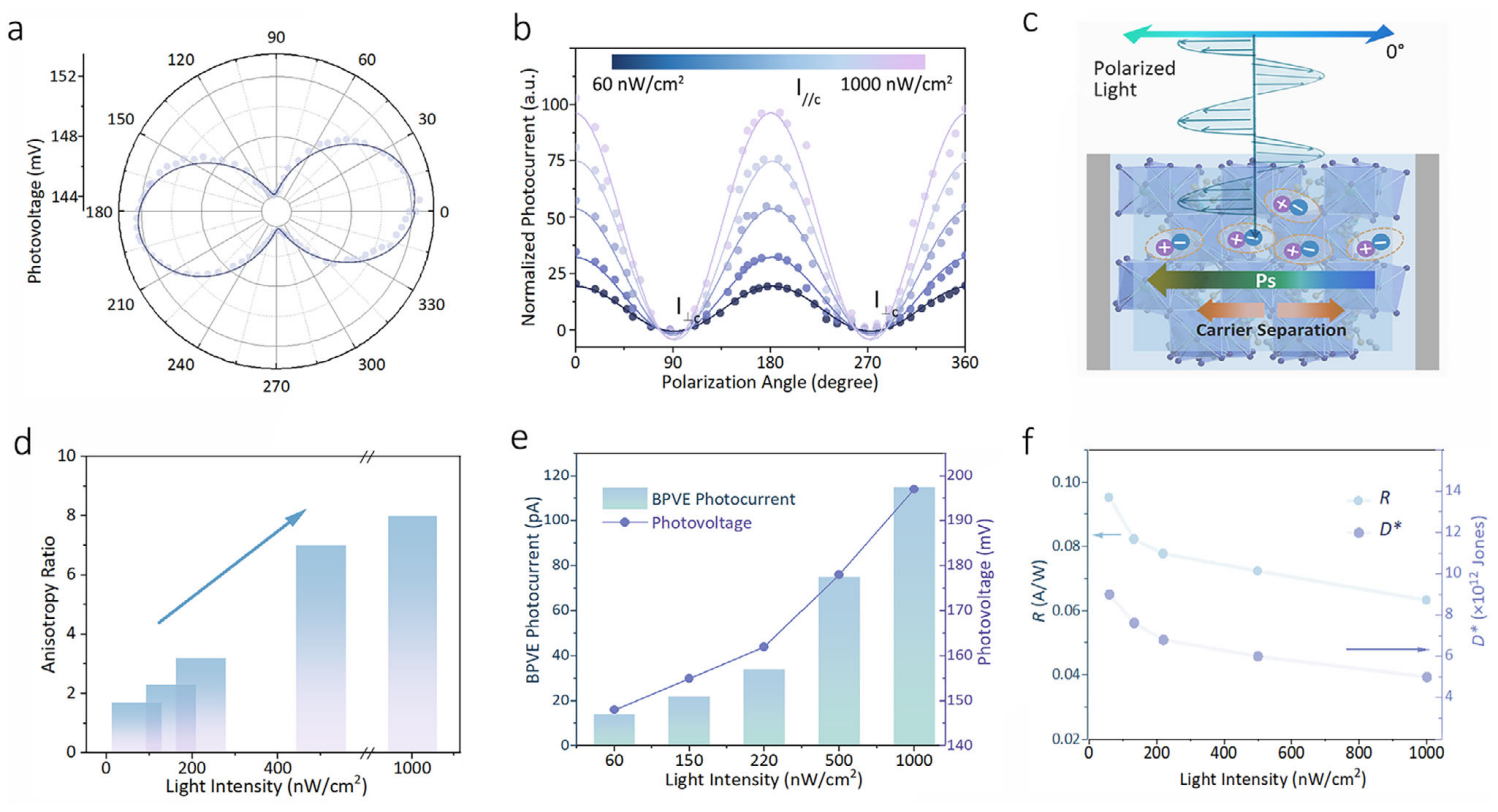
(a) Polarization‐dependent photovoltage of the (4‐AMP)PbBr_4_ crystal devices. (b) Photocurrent of the detectors as a function of the rotation angle under the illumination of a 377 nm laser at 0 V bias. (c) Mechanism of the self‐powered polarization‐sensitive photodetectors using the polar (4‐AMP)PbBr_4_ crystals. (d) Anisotropy ratio of the present device under different light intensities. (e) BPVE photocurrent and photovoltage of the (4‐AMP)PbBr_4_ crystal‐based self‐powered polarization‐sensitive photodetectors under 60 nW/cm^2^ light illumination. (f) D* and *R* of the detectors.

The high polarization sensitivity of the (4‐AMP)PbBr_4_ crystal detectors can be explained by the inherent light polarization dependence of BPVE (Figure 4d). The photocurrent produced by the BPVE can be described by the equation *J*_c_ = *I*β_31_sin^2^(*θ*) + *I*β_33_cos^2^(*θ*), where *J*_c_ is the photocurrent density along the polar axis and *θ* is the polarization angle. When the incident polarized light is perpendicular to the polar axis of (4‐AMP)PbBr_4_, the β_31_ value is ∼0. This indicates the coupling between the spontaneous polarization and optical field is weak, resulting in negligible BPVE and low *I*_⊥c_. In contrast, when the polarized light is incident parallel to the polar axis, the light–matter interaction between the incident UV light and spontaneous polarization generates significant BPVE and a large photocurrent *I*_IIc_. Therefore, self‐powered UV photodetectors based on the polar (4‐AMP)PbBr_4_ crystals are highly polarization sensitive even exposing to a weak polarized light. Moreover, photocurrent and photovoltage of the (4‐AMP)PbBr_4_ crystal self‐powered detectors under 377 nm illumination at the polarization angle at 0° is shown in Figure 4e. The BPVE photocurrent under 60 nW/cm^2^ weak light illumination exhibits good stability and reproducibility, supporting its weak light detection ability (Figure S12). Compared with the devices working under external bias voltages, the power‐law fitting of BPVE photocurrent as a function of light intensity yields a smaller exponent α = 0.76 (Figure S13). This behavior arises from less efficient carrier separation and transport driven solely by the built‐in electric field. Furthermore, responsivity (*R*) and detectivity (D*), as two important parameters, are introduced to evaluate the detection performance of the (4‐AMP)PbBr_4_ crystal polarization‐sensitive photodetectors. As shown in Figure 4f, the *R* value of 100 mA/W is achieved under a light density of 60 nW/cm^2^. The highest D* value of 8.8 × 10^12^ Jones is comparable to other excellent photodetectors of hybrid perovskites (Figure S14 for the calculation details). In addition, response time is another key figure‐of‐merit for photodetectors, consisting of rise time and fall time. The rise time is defined as the duration for photocurrent to increase from 10% to 90% of the saturated signal, while the fall time describes the period for photocurrent to drop from 90% to 10% of the peak response upon switching off the incident light. Based on the definition of response speed, the rise and decay times of the (4‐AMP)PbBr_4_ crystal polarization‐sensitive photodetector in a self‐powered mode are estimated to be around 0.23 and 0.3 ms, respectively (Figure S15). The large discrepancy between the response time and TRPL lifetime indicates interfacial loss and massive RC delays of the devices. To further improve the detector performance, optimization of the device structure, interfacial transport, and crystal thickness is required.

## 3 Conclusion

In this paper, we have shown that exploiting polar OIHP crystals can enable self‐powered UV photodetectors with low detection limit and high polarization sensitivity. The (4‐AMP)PbBr_4_ we used here exhibits a polar structure of *P*ca2_1_ and features distinctive BPVE with a photovoltage of 0.15 V. The impressive BPVE enables (4‐AMP)PbBr_4_‐based photodetectors operating without external energy supply and being highly polarized sensitive. Under self‐powered mode, photodetectors using (4‐AMP)PbBr_4_ crystals show pronounced photoresponse to ultraweak UV light and present polarization‐dependent photocurrent with a large anisotropy ratio of 8 at 1 µW/cm^2^ and even 1.8 at 60 nW/cm^2^. The weak light detection ability of the present devices can be ascribed to the ultralow dark current under self‐powered mode. The achievement of self‐powered UV polarization‐sensitive photodetectors showing the ability of weak light detection in polar 2D HOIPs may open an avenue for polarized light imaging and other advanced optoelectronic applications.

## Author Contributions

**Jianbo Wu**: software, data curation. **Xinyuan Zhang**: conceptualization, investigation, methodology, software, data curation, project administration, writing – review and editing, writing – original draft. **Wai‐Yeung Wong**: writing – review and editing, resources, supervision.

## Conflicts of Interest

The authors declare no conflicts of interest.

## Supporting information




**Supporting File**: advs76653‐sup‐0001‐SuppMat.doc.

## Data Availability

The data that support the findings of this study are available from the corresponding authors upon reasonable request.
