# Supplementary material for: Exploiting Bulk Photovoltaic Effect in a Polar Hybrid Perovskite Towards Self‐Powered Detection of Weak Ultraviolet Polarized Light
Source: Adv Sci (Weinh). 2026 Jul 23:e76653. Online ahead of print. doi: 10.1002/advs.76653 (PMC13393280; doi:10.1002/advs.76653)
Supplement: Supplementary file 1 — Supporting File: advs76653‐sup‐0001‐SuppMat.doc. [file ADVS-9999-e76653-s001.doc]

**Exploiting Bulk Photovoltaic Effect in a Polar Hybrid Perovskite towards Self-Powered Detection of Weak Ultraviolet Polarized Light**

*Xinyuan Zhang, Jianbo Wu, and Wai-Yeung Wong**

**Table of Contents**

1.Experimental Section 3

2. Results and Discussion

Figure S1 6

Figure S2 6

Figure S3 7

Figure S4 7

Figure S5 8

Figure S6 8

Figure S7 9

Figure S8 9

Figure S9 10

Figure S10 10

Figure S11 11

Figure S12 11

Figure S13 12

Figure S14 12

Figure S15 13

Table S1 14

Table S2 15

3. References 17

**1. Experimental Section**

**Materials:** 4-(Aminomethyl)-piperidine solution (4-AMP), PbBr_2_, and 48% hydrobromic acid solution (HBr) were purchased from Aladdin (Shanghai, China). All chemicals and solvents were used as received without further purification.

**Synthesis of (4-AMP)PbBr_4_ crystals:** (4-AMP), and PbBr_2_ with stoichiometric ratio of 4: 1 were dissolved in 30 ml HBr. The solution was maintained at a temperature of 70 °C and stirred for 3 hours to ensure the complete dissolution of the solutes. Next, the solution was cooled to 35 °C (3 °C/day) for the crystal growth. After about 13-days growth, large-sized crystals could be obtained.

**Single Crystal Characterization**: Single-crystal XRD was conducted on a Bruker D8 diﬀractometer with graphite-monochromatized Mo K*α* radiation at 200 K.

**Powder X-ray diffraction**: Powder X-ray diffraction measurement was performed on a Rigaku MiniFlexⅡ diffractometer at an atmospheric environment. The diffraction patterns were collected in the 2θ range of 5°–45° with a step size of 0.5°/min.

**Scanning Electron Microscope Measurement (SEM)**: The SEM image was collected on SU 8010 field-emission scanning electron microscope operated at 3 kV.

**Absorption Spectrum Measurement:** Absorption spectrum of (4-AMP)PbBr_4_ was performed on a Perkin-Elmer Lambda 900 UV–Vis–NIR spectra photometer at room temperature, in which BaSO_4_ was used as the 100% reflectance reference.

**Bandgap Calculation:** The optical bandgap of (4-AMP)PbBr_4_ was derived from the Tauc equation: (*αhυ*) = *B*(*hυ-E_g_*)^n^, where *α, hυ, E_g_* and *B* correspond to absorption coefficient, photon energy, optical bandgap and constant, respectively.

**Photoluminescence Spectra Measurements**: Emission spectra of (4-AMP)PbBr_4_ were performed on an Edinbergh FLS980 fluorescence spectrometer. The lifetime of (4-AMP)PbBr_4_ was measured on an Edinburgh FLS980 fluorescence spectrometer using a picosecond pulsed diode laser. The dynamics of emission decay were monitored by using the FLS980’s time-correlated single-photon counting capability (1,024 channels; 1 µs window) with data collection for 10,000 counts in the maximum channel.

**Trap Density Measurement:** In the device, thickness of the crystal is about 1 mm to test the carrier transport properties. The trap density was calculated according to the calculation based on the following equation: *n_trap_* = *2εε_0_V_TFL_/eL^2^*, where *ε* is the relative dielectric constant, *ε*_0_ is the vacuum permittivity, *L* is the thickness of the material and *e* represents the element charge

**Calculation for carrier mobility and carrier diffusion length:** The carrier mobility can be calculated using the Mott’s SCLC theory: *J_D_ = 9εε_0_µV_b_^2^/8L^3^,* where *J_D_, ε, ε_0_, µ, V_b_,* and *L* are the dark current density, the relative dielectric constant, the vacuum permittivity, the mobility of the crystal, applied voltage, and the thickness of the crystal, respectively. The carrier diffusion length (*L_D_*) can be obtained by the following equation: *L_D_* = (*Dτ*)^1/2^ = [(*k_B_Tµτ*)/*e*]^1/2^*,* in which *D* is the diffusion coefficient, *τ* is the carrier lifetime, *k_B_* is the Boltzmann’s constant and *T* is the sample temperature.

**Device Preparation.** Photodetectors for both unpolarized- or polarized-light detection were fabricated using the grown (4-AMP)PbBr_4_ crystals with dimensions of 2 × 1 × 1 mm^3^. Silver (Ag) electrodes are deposited perpendicular to the *c*-axis of (4-AMP)PbBr_4_ crystals. Crystal-based electrodes deposited with silver conducting paste were used for electrical measurement. The electrode materials were proven not to have any obvious influence on the optoelectronic properties. The channel between neighboring electrodes had a width of 0.2 mm and a length of 1 mm.

**Visible Light Detectors**: The current vs voltage (*I–V*) and voltage vs time (*V-t*) measurements were conducted using a high precision electrometer (Keithley 6517B). *I–V* and *V-t* tests were collected under the 377 nm continuous-wave laser (ITC4001). The incident light intensity was measured by a light power meter. The temperature during measurements was controlled at 290 K using a Linkam TS1500 heating stage. The effective illuminated device area was estimated to be 0.2 mm^2^.

**Polarization-sensitive Photodetectors**: The current vs voltage (*I–V*), photocurrent vs time (*I–t*), and voltage vs time (*V–t*) with light on and off (measured at zero bias) were measured using a high precision electrometer (Keithley 6517B). *I–V* and *I–t* tests were collected under the 377 nm continuous-wave laser (ITC4001). The incident light intensity was measured by a light power meter. The temperature during measurements was controlled at 298 K using a Linkam TS1500 heating stage. The responsivity (*R*) and detectivity (*D**) can be calculated by the following equations. *R* is given by the equation: R = *I*_ph_/*P*_in_ = *I*_light_*-I*_dark_/*P*_in_, where the *I*_ph_ is the photocurrent generated with incident light illumination (calculated by subtracting current in the dark from the current under the light illumination, namely, *I*_light_*-I*_dark_), and *P*_in_ stands for the incident light power intensity on the effective area of the device. *D** is defined as: *D** = *A^1/2^/NEP*, where *A* is the illuminated area of the photodetector (the unit is cm^2^), *NEP* is the noise equivalent power and calculated by NEP = noise/R, where *R* is the responsivity. The noise of 2D materials photodetectors primarily includes 1/*f* noise, shot noise, and thermal noise. The 1/*f* noise usually plays a role in low frequency and is much less than shot noise and thermal noise with several orders of magnitude. Besides, the shot noise is much smaller than the thermal noise at small or zero bias, and gradually play a significant role in total noise with increasing biases. In the case of our devices, the thermal noise is the main noise source due to the zero-bias operation condition. Thus, the *D** could be calculated by the formula: *D** = *R*/(4*k_B_T*/*R’A*)^1/2^, where *R’* is the resistance, *k*_B_ is the [Boltzmann constant](http://dict.youdao.com/w/boltzmann constant/" \l "keyfrom=E2Ctranslation), *T* is the temperature. In addition, the rise and decay times are defined as the time necessary to reduce/or increase the photoresponse from 90% to 10%.

**2. Results and Discussion**


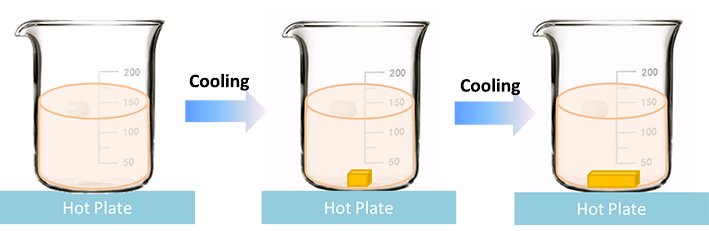


**Figure S1.** Growth of (4-AMP)PbBr_4_ crystals via a temperature-cooling solution method.


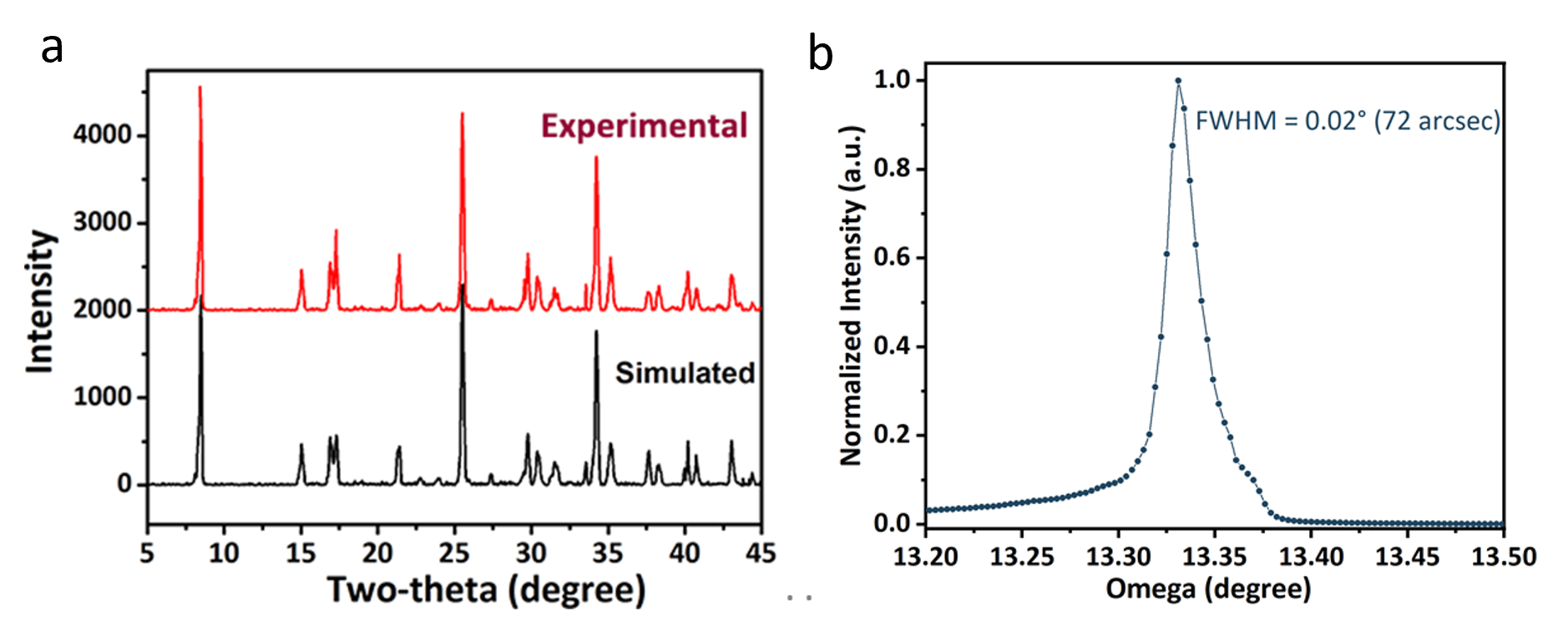


**Figure S2.** (a) PXRD patterns of the 2D (4-AMP)PbBr_4_. (b) X-ray rocking curves of the (4-AMP)PbBr_4_ crystal.


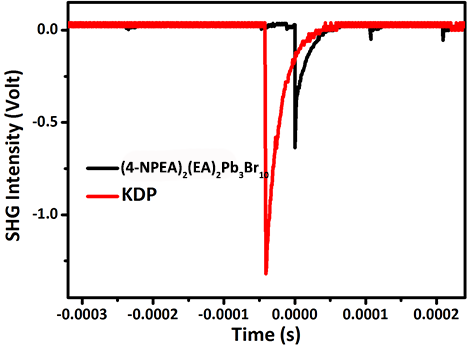


**Figure S3.** SHG signal of the 2D (4-AMP)PbBr_4_.


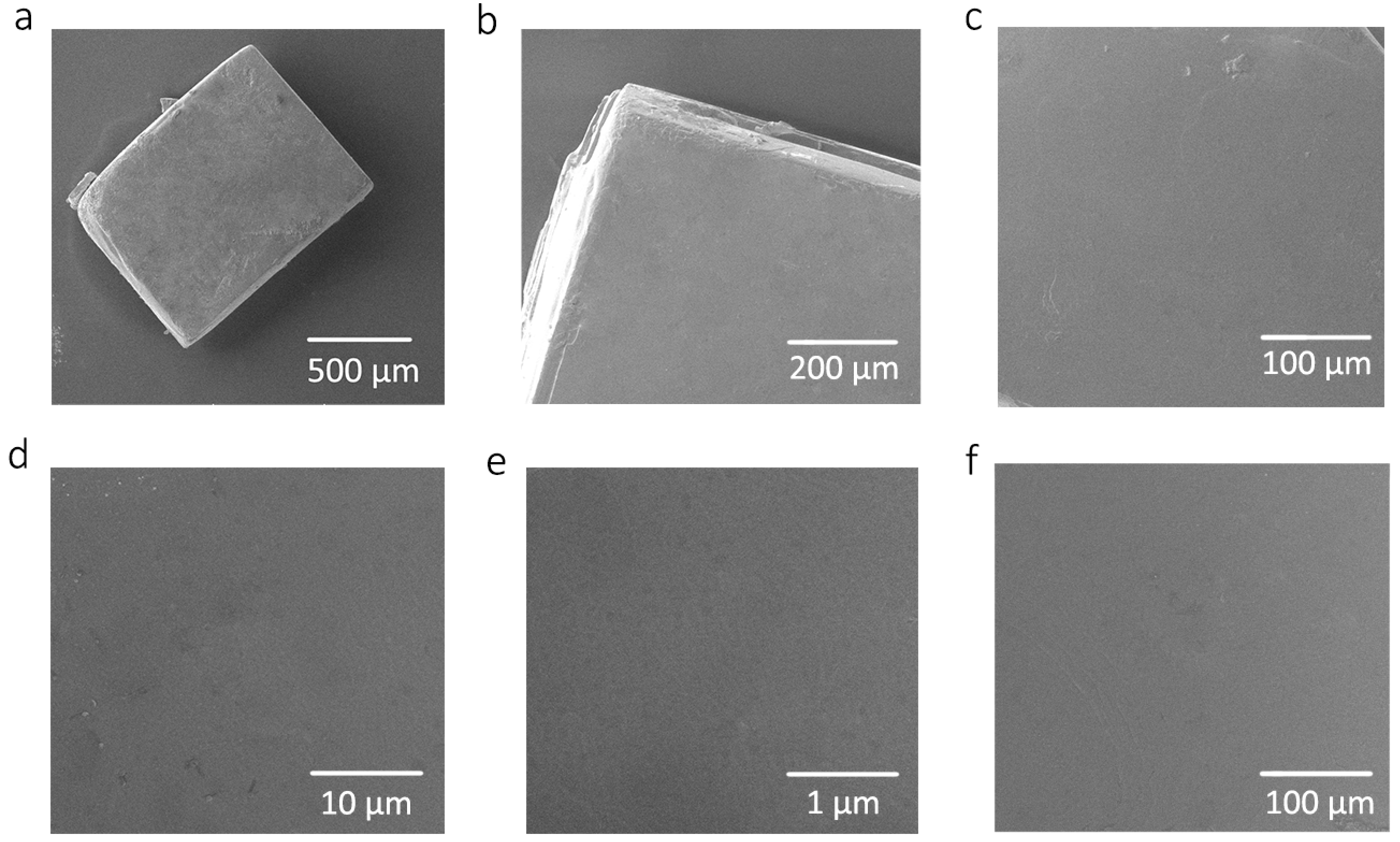


**Figure S4.** SEM images of the bulk crystals with scale bar of (a) 500 µm, (b) 200 µm, (c) 100 µm, (d) 10 µm, and (e) 1 µm. (f) SEM images of the newly exfoliated thin flakes.

.


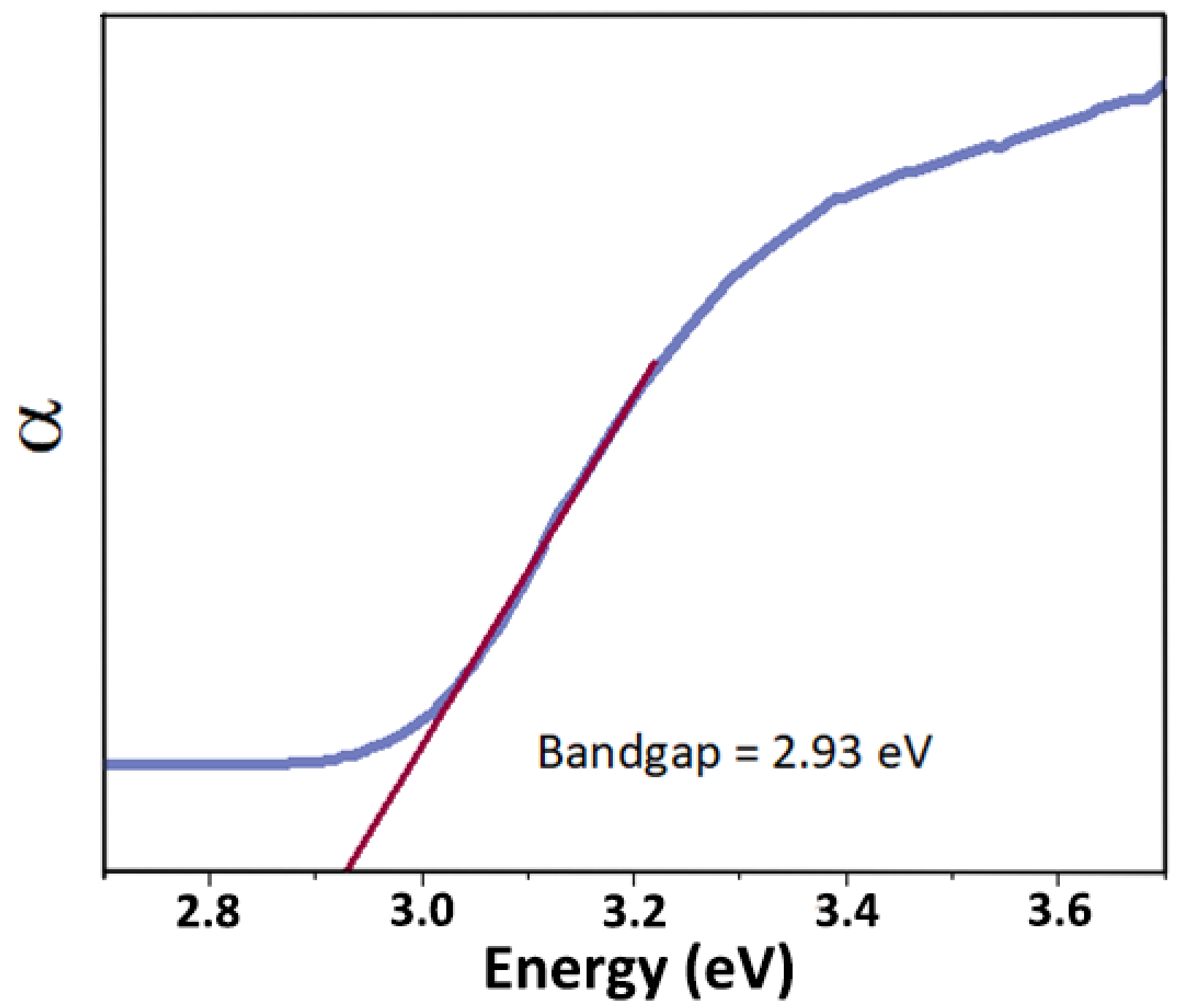


**Figure S5.** Bandgap of the (4-AMP)PbBr_4_.


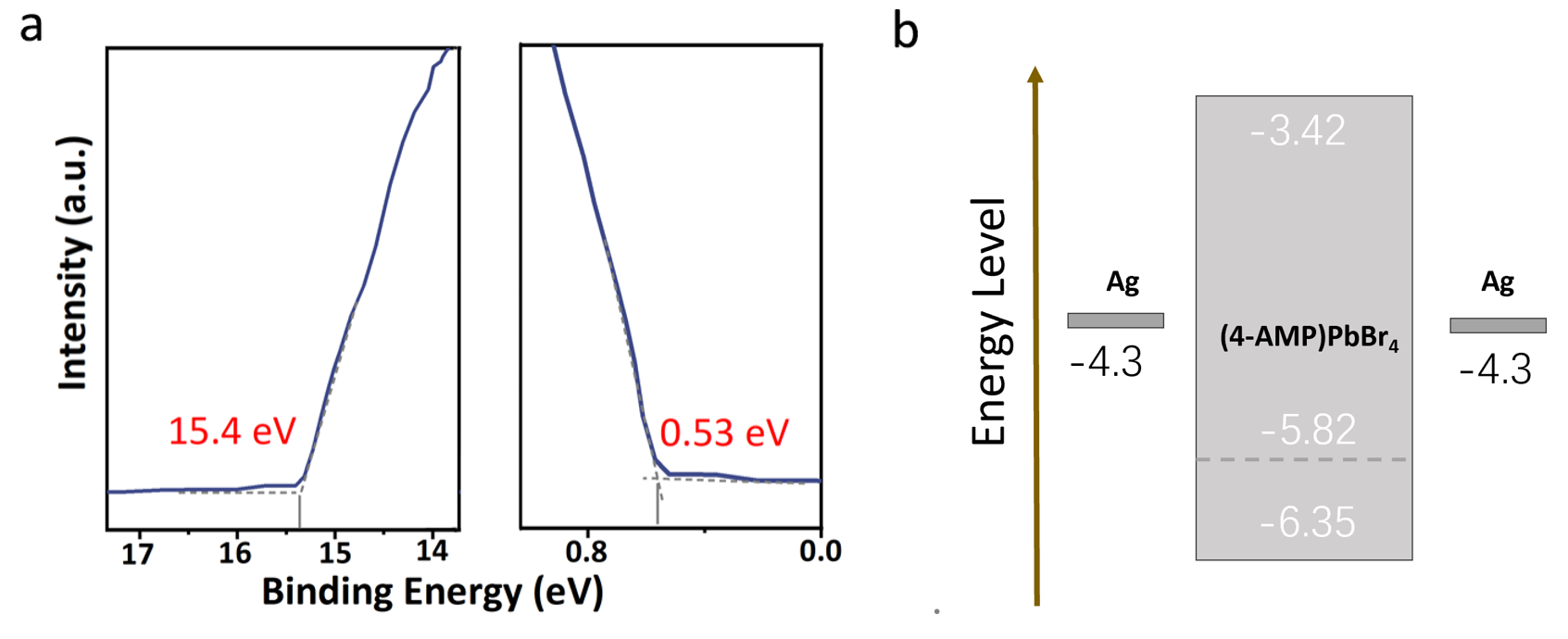


**Figure S6.** (a) The UPS of 2D (4-AMP)PbBr_4_. (b) The energy band alignment of the (4-AMP)PbBr_4_-based device.


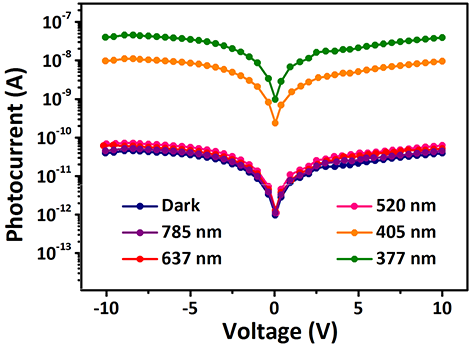


**Figure S7.** Photoresponse of the detectors using (4-AMP)PbBr_4_ crystals.


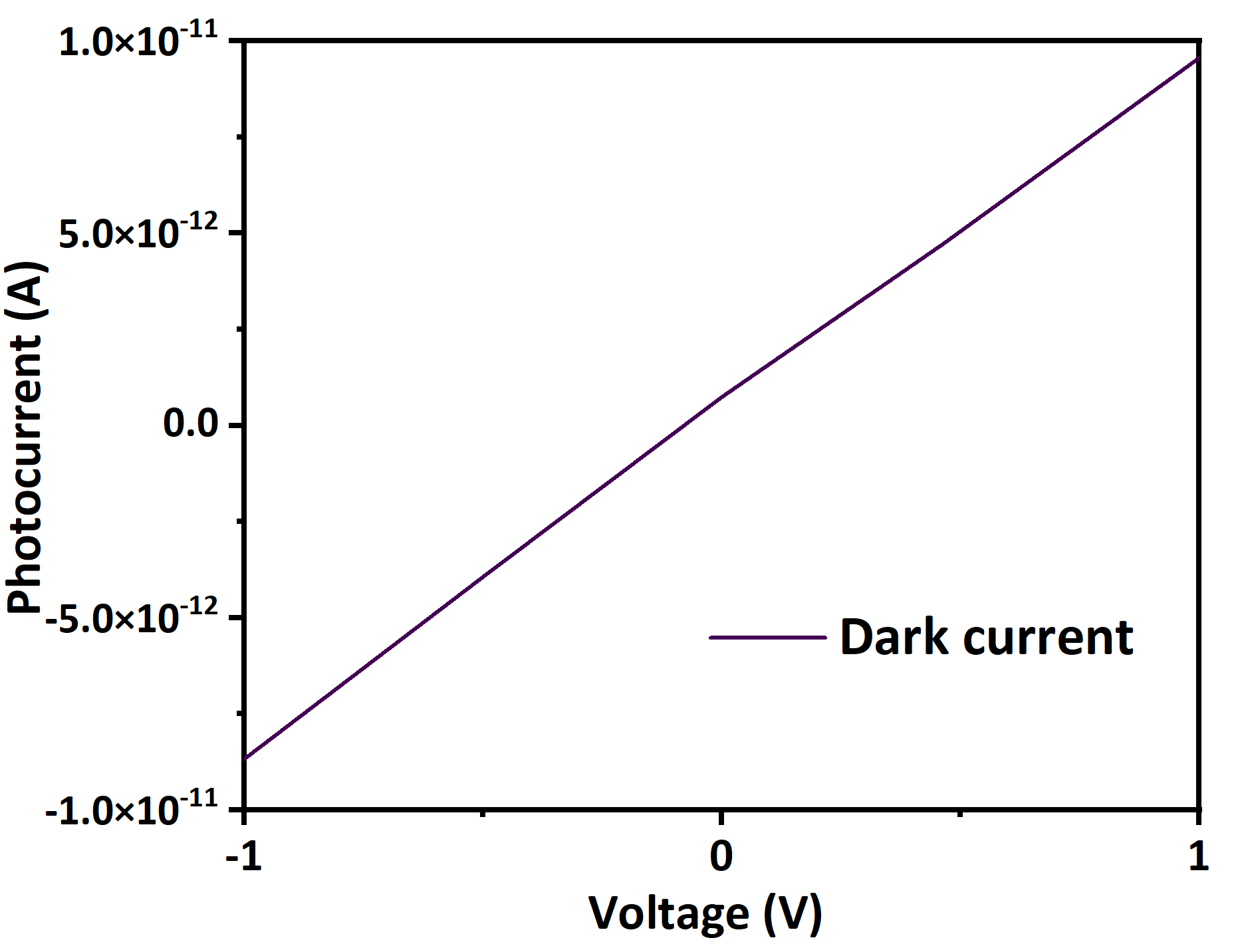


**Figure S8.** The magnified linear-scale *I-V* curve of the dark current from -1 to 1 V.


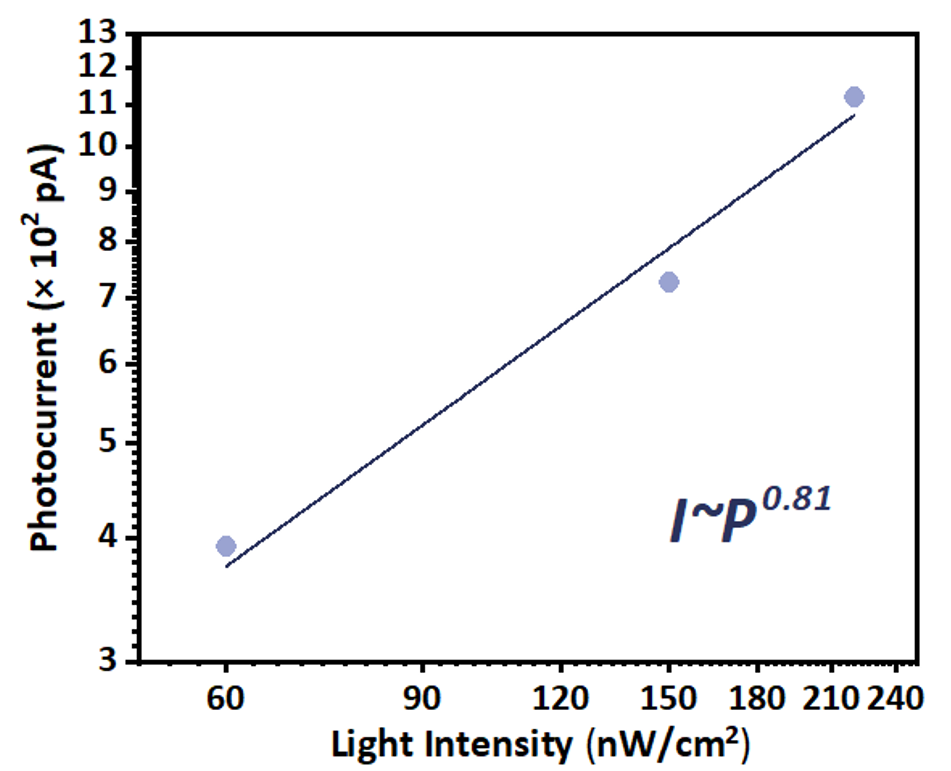


**Figure S9.** The power-law fitting of photocurrent of the devices under external bias of 10 V.


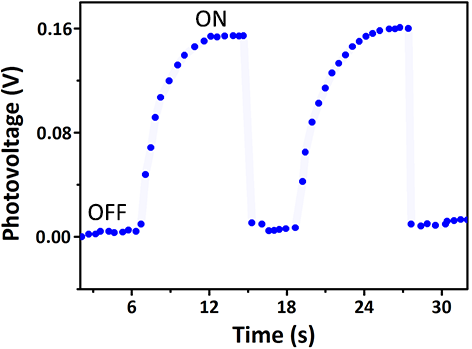


**Figure S10.** *V-t* switching cycles of the (4-AMP)PbBr_4_ crystal-based photodetectors.


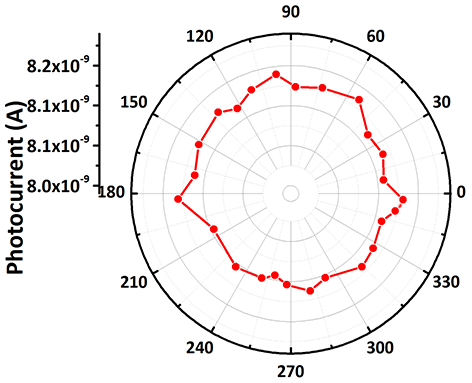


**Figure S11.** Angle-dependent photocurrent of the (4-AMP)PbBr_4_ crystal-based photodetectors along the *a*-axis of the (4-AMP)PbBr_4_ crystal.


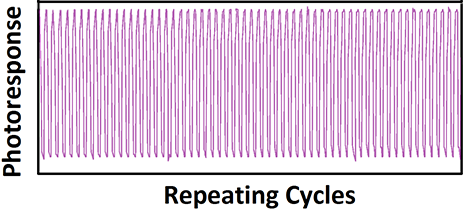


**Figure S12.** The on/off switching cycles of the (4-AMP)PbBr_4_ crystal polarized light detectors at zero bias.


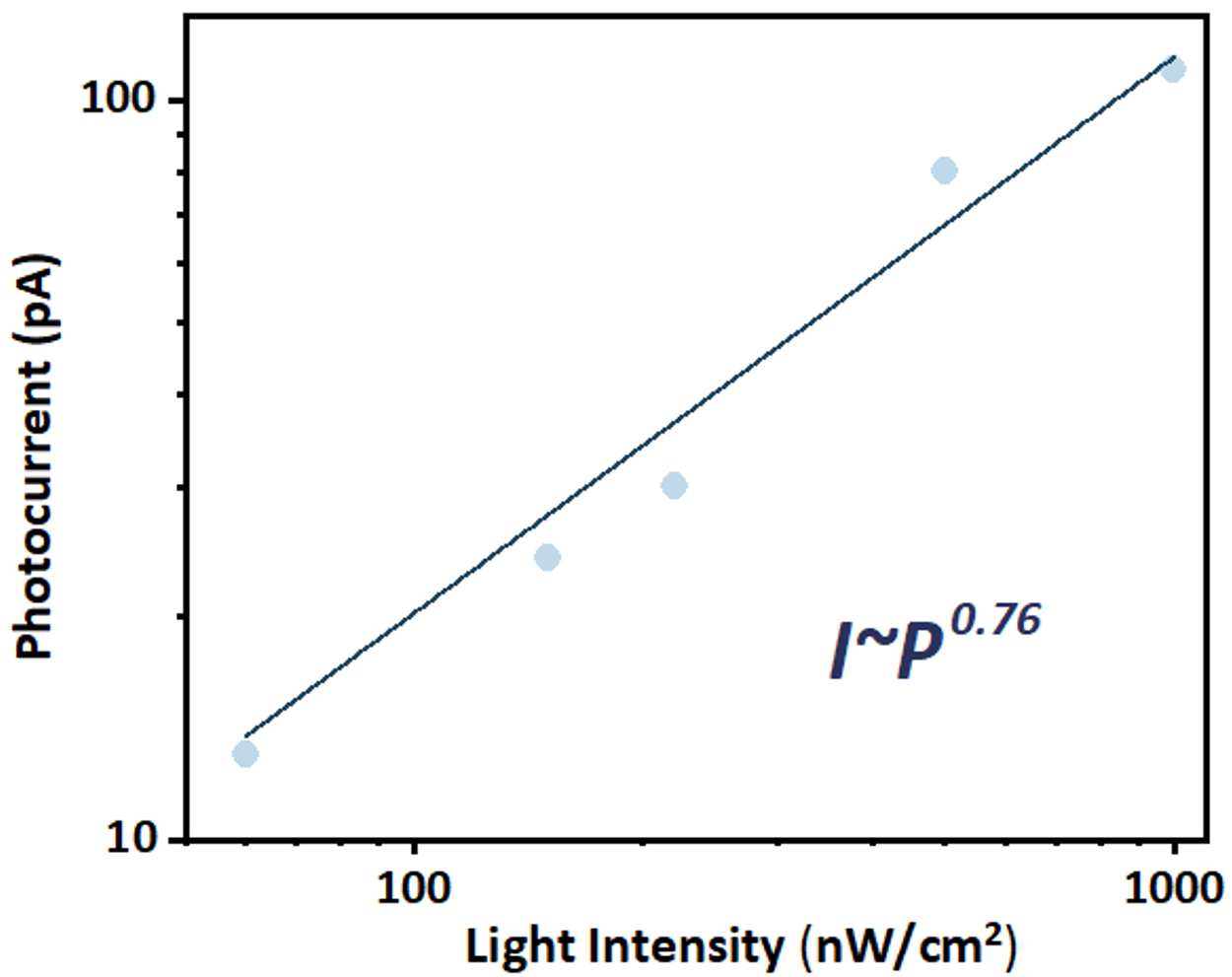


**Figure S13.** The power-law fitting of BPVE photocurrent of the devices.


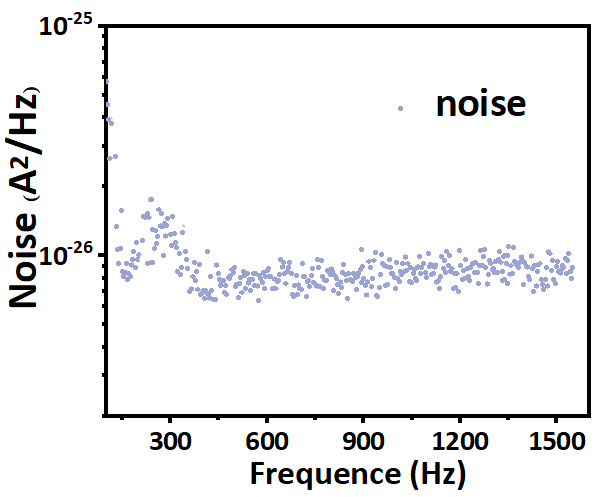


**Figure S14.** The current noise power spectra of the photodetectors operating at 0 V bias.


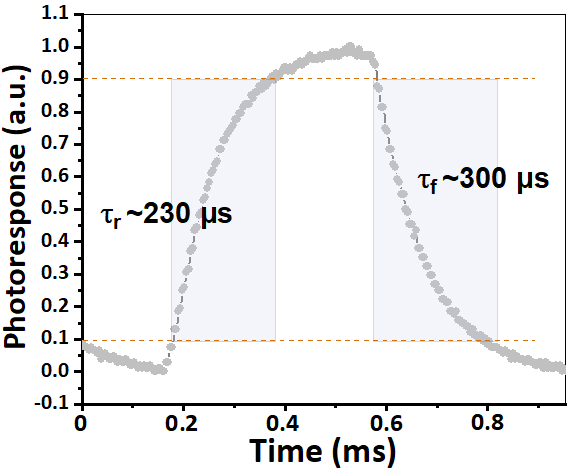


**Figure S15.** Response time of the (4-AMP)PbBr_4_ crystal-based self-powered polarization-sensitive photodetectors.

**Table S1** Comparison of the FWHM of the PXRD patterns of the (4-AMP)PbBr_4_ crystals with other reported crystals

| **Materials** | **FWHM (degree)** | **Ref.** |
| --- | --- | --- |
| (4-AMP)PbBr_4_ crystal | 0.02 | This work |
| Cs_3_Cu_2_I_5_ crystal | ~0.01 | [1] |
| Cs_3_Cu_2_I_5_ wafer | 0.049 | [2] |
| (PEA)_2_PbBr_4_ crystal | 0.051 | [3] |
| BA_2_PbBr_4_ crystal | 0.063–0.076 | [4] |
| MAPbI_3_ crystal | 0.016 | [5] |
| CsPbBr_3_ crystal | 0.043 | [6] |

.

**Table S2** Comparison of (4-AMP)PbBr_4_ crystal polarization-sensitive photodetector with other reported devices.

|  | Detectors | λ (nm) | Anisotropy  Ratio | Condition | Ref |
| --- | --- | --- | --- | --- | --- |
| 2D  material | (AA)_2_(EA)_2_Pb_3_Br_10_ | 405 | ~15 | Self-driven | [7] |
|  | (BPA)_2_PbBr_4_ | 377 | ~6.8 | Self-driven | [8] |
|  | (FPEA)_2_(MA)Pb_2_I_7_ | 520 | ~1.5 | Self-driven | [9] |
|  | (BA)_2_MAPb_2_Br_7_ | 405 | ~2 | 10 V | [10] |
|  | BA_2_CsPb_2_Br_7_ | 405 | ~1.5 | 10 V | [11] |
|  | (*i*BA)_2_(MA)Pb_2_I_7_ | 637 | ~1.23 | 10 V | [12] |
|  | *i*BAPbI_4_ | 552 | 1.56 | -5 V | [13] |
|  | (FPEA)_2_PbI_4_ | 520 | ~2.1 | 10 V | [14] |
|  | [CH(NH_2_)_2_][C(NH_2_)_3_]PbI_4_ | 515 | ~2 | -3 V | [15] |
|  | (CBA)_2_CsPb_2_Br_7_ | 405 | ~2.7 | Self-driven | [16] |
|  | BDAEA_2_Pb_3_Br_10_ | 532 | ~2.4 | Self-driven | [17] |
|  | (MBA)_2_(MA)_2_Pb_3_I_10_ | 520 | ~13.8 | Self-driven | [18] |
|  | (BA)_2_CsAgBiBr_7_ | 405 | ~1.38 | Self-driven | [19] |
|  | (BA)_2_(MA)_2_Pb_3_I_10_ | 637 | ~1.96 | Self-driven | [20] |
|  | (i-PA)_2_CsAgBiBr_7_ | 405 | ~1.35 | 10 V | [21] |
|  | GeSe | 532 | ~1.09 | 2 V | [22] |
|  | GeAs_2_ | 532 | ~2 | 1 V | [23] |
|  | BP | 1200 | ~3.5 | 0.1 V | [24] |
|  | PdSe_2_ | 4600 | ~2.06 | Self-driven | [25] |
| Nanowire | CsPbBr_3_ | 470 | ~2.6 | 5 V | [26] |
|  | MAPbI_3_ | 530 | ~1.3 | 1 V | [27] |
|  | InP | 514 | ~10 | 0.05 V | [28] |
|  | KNb_3_O_8_ | 254 | ~1.6 | 5 V | [29] |
| Heteros-  tructure | Graphene/PdSe_2_/Ge | 650 | ~112 | Self-driven | [30] |
|  | SbI_3_/SbO_3_ | 450 | ~3 | 8 V | [31] |
|  | BP/hBN | mid-IR | ~6.4 |  | [32] |
|  | hBN/(b-As_x_P_1-x_)/hBN | 5000 | ~14 | 1 V | [33] |
|  | BP/InSe | 633 | ~10.76 | Self-driven | [34] |
|  | BPt/MoS_2_/BPb | 3500 | ~22 |  | [35] |
|  | PdSe_2_/FA_1-x_Cs_X_PbI_3_ | 808 | ~6 | Self-driven | [36] |
|  | (4-AMP)(MA)_2_Pb_3_Br_10_  /MAPbBr_3_ | 405 | ~17 | Self-driven | [37] |
|  | (BLA)_2_CsAgBiBr_7_/Cs_2_AgBiBr_6_ | 405 | ~4 | Self-driven | [38] |

**3. References**

[1] Q. Yao, J. Li, X. Li, Y. Ma, H. Song, Z. Li, Z. Wang, X. Tao, *Adv. Mater.* **2023***, 35,* 2304938.

[2] W. Wu, J. Zhang, C. Liu, J. Zhang, H. Lai, Z. Hu, H. Zhou, *Adv. Sci.* **2024***, 11,* 2410303.

[3] B. Yang, X. Ouyang, X. Zhao, J. Su, Y. Li, S. Zhang, X. Ouyang, *InfoMat.* **2025***,7,* e12648.

[4] X. Xu, Y. Wu, Y. Zhang, X. Li, F. Wang, X. Jiang, S. Wu, S. Wang, *Energy Environ. Mater.* **2024***, 7,* e12487.

[5] D. Liu, Y. Zheng, X. Sui, X. Wu, C. Zou, Y. Peng, X. Liu, M. Lin, Z. Wei, H. Zhou, Y. Yao, S. Dai, H. Yuan, H. Yang, S. Yang, Y. Hou, *Nat. Commun.* **2024***, 15,* 2390.

[6] M. Chen, Y. Yuan, Y. Liu, D. Cao, C. Xu, *RSC Adv.* **2022***, 12,* 14838.

[7] Y. Peng, X. Liu, Z. Sun, C. Ji, L. Li, Z. Wu, S. Wang, Y. Yao, M. Hong, J. Luo, *Angew. Chem. Int. Ed.* **2020**, *59*, 3933.

[8] C. Ji, D. Dey, Y. Peng, X. Liu, L. Li, J. Luo, *Angew. Chem. Int. Ed.* **2020**, *59*, 18933.

[9] X. Hu, H. Xu, Y. Liu, L. Lu, W. Guo, S. Han, J. Luo, Z. Sun, *J. Phys. Chem. Lett.* **2022**, *13*, 6017.

[10] L. Li, X. Liu, Y. Li, Z. Xu, Z. Wu, S. Han, K. Tao, M. Hong, J. Luo, Z. Sun, *J. Am. Chem. Soc.* **2019**, *141*, 2623.

[11] J. Wang, Y. Liu, S. Han, Y. Ma, Y. Li, Z. Xu, J. Luo, M. Hong, Z. Sun, *Sci. Bull.* **2021**, *66*, 158.

[12] Y. Liu, Z. Wu, X. Liu, S. Han, Y. Li, T. Yang, Y. Ma, M. Hong, J. Luo, Z. Sun, *Adv. Opt.* *Mater*. **2019**, *7*, 1901049.

[13] L. Li, L. Jin, Y. Zhou, J. Li, J. Ma, S. Wang, W. Li, D. Li, *Adv. Opt.* *Mater*. **2019**, *6*, 1900988.

[14] M. Li, S. Han, B. Teng, Y. Li, Y. Liu, X. Liu, J. Luo, M. Hong, Z. Sun, *Adv. Opt.* *Mater*. **2020**, *6*, 2000149.

[15] C. Fang, M. Xu, J. Ma, J. Wang, L. Jin, M. Xu, D. Li, *ACS Nano* **2020**, *20*, 2339.

[16] L. Wang, C. Wu, Z. Xu, H. Wu, X. Dong, T. Chen, J. Liang, S. Chen, J. Luo, L. Li, *Small* **2024**, *20*, 2310166.

[17] X. Yang, B. Zhou, M. Guo, Y. Liu, R. Cong, L. Li, W. Wu, S. Wang, L. Guo, C. Pan, Z. Yang, *Adv. Sci.* **2025**, *12,* 2414422.

[18] H. Ni, H. Xu, Y. Liu, X. Zeng, W. Guo, P. Zhu, Z. Zhao, H. Rong, J. Luo, Z. Sun, *Adv. Optical Mater.* **2024**, *12*, 2401011.

[19] Q. Yin, J. Wu, H. Ye, H. Li, Z. Zhu, C. Zhang, L. Xu, Z. Han, J. Luo, *Adv. Optical Mater.* **2025**, *13*, 2403183.

[20] Z. Xu, X. Dong, L. Wang, H. Wu, Y. Liu, J. Luo, M. Hong, L. Li, *J. Am. Chem. Soc.* **2023**, *145*, 1524.

[21] Y. Li, T. Yang, Z. Xu, X. Liu, X. Huang, S. Han, Y. Liu, M. Li, J. Luo, Z. Sun*, Angew. Chem. Int. Ed.* **2020**, *59*, 3429.

[22] X. Wang, Y. Li, L. Huang, O. Jiang, L. Jiang, H. Dong, Z. Wei, J. Li, W. Hu, *J. Am. Chem. Soc.* **2017**, *139*, 14976.

[23] L. Li, P. Gong, D. Sheng, S. Wang, W. Wang, X. Zhu, X. Shi, F. Wang, W. Han, S. Yang, K. Liu, H. Li, T. Zhai, *Adv. Mater.* **2018**, *30*, e1804541.

[24] S. Wu, Y. Chen, X. Wang, H. Jiao, Q. Zhao, X. Huang, X. Tai, Y. Zhou, H. Chen, X. Wang, S. Huang, H. Yan, T. Lin, H. Shen, W. Hu, X. Meng, J. Chu, J. Wang, *Nat. Nanotech.* **2015**, *10*, 707.

[25] M. Dai, C. Wang, M. Ye, S. Zhu, S. Han, F. Sun, W. Chen, Y. Jin, Y. Chua, Q. Wang, *ACS Nano* **2022**, *16*, 295.

[26] J. Feng, X. Yan, Y. Liu, H. Gao, Y. Wu, B. Su, L. Jiang, *Adv. Mater.* **2017**, *29*, 1605993.

[27] L. Gao, K. Zeng, J. Guo, C. Ge, J. Du, Y. Zhao, C. Chen, H. Deng, Y. He, H. Song, G. Niu, J. Tang, *Nano Lett.* **2016**, *16*, 7446.

[28] J. Wang, M. Gudiksen, X. Duan, Y. Cui, C. M. Lieber, *Science* **2001**, *293*, 1455.

[29] Y. Ping, H. Long, H. Liu, C. Chen, N. Zhang, H. Jing, J. Lu, Y. Zhao, Z. Yang, W. Li, F. Ma, X. Fang, Z. Wei, H. Xu, *Adv. Funct. Mater.* **2022**, *32,* 2111673.

[30] D. Wu, J. Guo, J. Du, C. Xia, L. Zeng, Y. Tian, Z. Shi, Y. Tian, L. Jian, Y. Tsang, J. Jie, *ACS Nano* **2019**, *13*, 9907.

[31] M. Xiao, H. Yang, W. Shen, C. Hu, K. Zhao, Q. Gao, L. Pan, L. Liu, C. Wang, G. Shen, H. Deng, H. Wen, Z. Wei, *Small* **2020**, *16*, e1907172.

[32] X. Chen, X. Lu, B. Deng, O. Sinai, Y. Shao, C. Li, S. Yuan, V. Tran, K. Watanabe, T. Taniguchi, D. Naveh, L. Yang, F. Xia, *Nat. Commun.* **2017**, *8*, 1672.

[33] S. Yuan, C. Shen, B. Deng, X. Chen, Q. Guo, Y. Ma, A. Abbas, B. Liu, R. Haiges, C. Ott, T. Nilges, K. Watanabe, T. Taniguchi, O. Sinai, D. Naveh, C. Zhou, F. Xia, *Nano lett.* **2018**, *18*, 3172.

[34] S. Zhao, J. Wu, K. Jin, H. Ding, T. Li, C. Wu, N. Pan, X. Wang, *Adv. Funct. Mater.* **2018**, *28*, 1802011.

[35] J. Bullock, M. Amani, J. Cho, Y. Chen, G. H. Ahn, V. Adinolfi, V. R. Shrestha, Y. Gao, K. B. Crozier, Y. Chueh, A. Javey, *Nat. Photon.* **2018**, *12*, 601.

[36] L. Zeng, Q. Chen, Z. Zhang, D. Wu, H. Yuan, Y. Li, W. Qarony, S. Lau, L. Luo, Y. Tsang, *Adv. Sci.* **2019**, *6*, 1901134.

[37] X. Zhang, L. Li, C. Ji, X. Liu, Q. Li, K. Zhang, Y. Peng, M. Hong, J. Luo, *Natl. Sci. Rev.* **2021**, *8*, nwab044.

[38] X. Zhang, Y. Yao, L. Liang, X. Niu, J. Wu, J. Luo, *Angew. Chem. Int. Ed.* **2022**, *51,* e202205939.
